# Relation of Lean Body Mass and Muscle Performance to Serum Creatinine Concentration in Hemodialysis Patients

**DOI:** 10.1155/2018/4816536

**Published:** 2018-06-04

**Authors:** Janez Vodičar, Jernej Pajek, Vedran Hadžić, Maja Bučar Pajek

**Affiliations:** ^1^University of Ljubljana, Faculty of Sport, Gortanova 22, 1000 Ljubljana, Slovenia; ^2^Department of Nephrology, University Medical Centre Ljubljana, Zaloška 2, 1525 Ljubljana, Slovenia

## Abstract

**Introduction:**

Serum creatinine concentration is an important uremic marker and predictor of survival in dialysis patients. This cross-sectional case-control study was made to quantitatively describe the relation between lean body mass (LBM), physical performance measures, and serum creatinine values.

**Methods:**

Ninety hemodialysis patients and 106 controls were measured by bioimpedance spectroscopy, handgrip strength, sit-to-stand test, and biochemical serum tests. Univariate and multivariate general linear models were used to analyze quantitative relations.

**Results:**

At univariate regression LBM accounted for 13.6% variability of serum creatinine concentration. In adjusted analyses with age, height, and body mass, LBM persisted as the only significant predictor of midweek predialysis serum creatinine concentration. Physical performance measures handgrip strength and sit-to-stand performance did not improve prediction of serum creatinine. With addition of serum urea concentration and residual diuresis the predictive value of the regression model improved to account for 45% of serum creatinine variability. Each kg of LBM was associated with 7.7 *μ*mol/l increase in creatinine concentration (95% CI 3.4-12.1, p=0.001).

**Conclusion:**

Bioimpedance derived LBM has a significant linear relation with predialysis serum creatinine concentrations. Hereby described quantitative relation should help clinicians to better evaluate observed creatinine concentrations of hemodialysis patients when bioimpedance derived LBM is available.

## 1. Introduction

Serum creatinine concentration may be regarded as the single most important laboratory parameter in routine nephrology clinical practice [1]. In end-stage renal disease (ESRD) patients, serum creatinine is not only used as an uremic marker but also as a predictor of nutritional status, muscle mass (which generates creatinine production), and survival [2–4]. Several authors have tried to predict lean body mass (LBM) or muscle mass from serum creatinine kinetics to facilitate the diagnosis of sarcopenia and malnutrition in dialysis patients [5–7]; however good quantitative data about the reverse relation, prediction of expected serum creatinine concentrations on the basis of estimated LBM, is needed as well. With the widespread use of bioimpedance technology in routine clinical dialysis setting it has become possible to predict the expected serum creatinine concentration on the basis of bioimpedance derived LBM. Clinical situations where this is of potential value are several. For example, in ESRD patients that have substantially low or high lean muscle mass there is a need to properly evaluate the discrepancy between observed and expected creatinine levels when deciding to start preparation for dialysis treatment (such as vascular access or peritoneal access placement). Similarly, in patients that progressively gain or lose lean weight on renal replacement therapy it is valuable to estimate their expected creatinine concentrations (on the basis of current LBM) to evaluate possible underdialysis (for example, when measured serum creatinine significantly exceeds predicted values).

Improvements in accessibility, reliability, and affordability of bioimpedance technology have enabled a widespread bedside use of this method in ESRD patients. Bioimpedance is used mainly to assess hydration status [8, 9] but (especially bioimpedance spectroscopy) also to assess body composition including the prediction of LBM [10]. Regular determination of body composition including LBM has now become a routine practice in dialysis setting [11]. With this, the option to predict serum creatinine concentration on the basis of bioimpedance derived LBM estimation has become possible.

The aim of this study was to quantitatively assess the relation of bioimpedance-assessed LBM to predialysis serum creatinine concentration in prevalent hemodialysis patients. Since there is a possibility that serum creatinine level depends not only on muscle mass but also on muscle quality as measured by handgrip strength [12], we wanted also to examine the relation between physical performance measures of upper and lower extremities with serum creatinine concentration.

## 2. Materials and Methods

### 2.1. Study Design and Participants

This study was performed as a secondary analysis of a cross-sectional case-control study recruiting a sample of renal disease-free control subjects and prevalent hemodialysis patients under care at 10 outpatient dialysis units [13]. Main outcome measure was a predialysis serum creatinine concentration with predictor covariates including demographic, clinical, body composition, and dialysis related parameters. Additionally, results of functional performance tests 10 repetitions of sit-to-stand test (STS-10) and handgrip strength (HGS) were measured and included in statistical analyses. Measurements were done in the period from July to December 2014. Selection criteria included age older than 18 years, being able to walk with or without additional support, and giving informed consent for participation. Exclusion criteria contained acute disease in the last 4 weeks before study start, active malignant disease or chronic infection, consequences of cerebrovascular accident, heart failure of NYHA stages 3-4, symptomatic angina pectoris Canadian Cardiovascular Society stages 2-4, chronic obstructive pulmonary disease stages 3 and 4, decompensated liver cirrhosis, symptomatic peripheral arterial obstructive disease, painful degenerative or inflammatory arthropathy with current use of analgesic therapy, and symptomatic psychiatric disease. Control subjects were recruited from a wide range of community settings (work sites, schools, nursing homes, and community centers for older adults). They should have had no history of renal disease or serum creatinine below 133 *μ*mol/l (1.5 mg/dl). Same comorbidity exclusion criteria as in dialysis cases were applied to controls. The study was approved by the Slovenian Medical Ethics Committee (document No. 125/05/14). All participants gave informed consent for inclusion in the study.

### 2.2. Research Protocol

The exact methodology and measurements description were described previously [13–16]. In short, physical performance tests were performed on nondialysis days to ensure safety for dialysis patients due to peaks of hypervolemia and hyperkalemia prior to dialysis sessions, increased risk of sudden death in the hours immediately prior to and the first hours after dialysis procedures [17], and increased levels of fatigue on dialysis days [18]. Demography, clinical information, and medical history were assessed by patient interview and medical documentation review and through contacts with attending nephrologists. Comorbidity was graded by Davies comorbidity score [19]. Residual diuresis was estimated by patients at home as an average of at least two days of urine output. Measurement protocol was started by anthropometric measurements (instruments by SiberHegner, Zurich, Switzerland), vital signs recordings, and bioimpedance spectroscopy using 3-compartment body composition analysis (Body Composition Monitor, Fresenius Medical Care, Bad Homburg, Germany). Here, estimated lean and fat mass in kg and overhydration of extracellular compartment in liters were measured.

Most recent midweek predialysis serum biochemistry values were obtained from routine dialysis surveillance tests. Serum creatinine was measured with calibrated kinetic Jaffe reaction traceable to isotope dilution mass spectrometry standard [20]. A single predialysis creatinine concentration value was taken for analyses on the basis of previous results showing high intraclass correlation coefficient and low intrapatient variance for this uremic marker [21]. Jamar hand dynamometer (Sammons Preston, Warrenville, IL, USA) was used to assess HGS engaging both hands three times and the best value of all attempts was taken as a result (in kg units). STS-10 time (in seconds) was measured as the time needed to perform rises from the chair of a standard height to the full leg extension and back to sitting position 10 times in a single attempt.

### 2.3. Statistical Analyses

Unadjusted between-group comparisons were made with independent samples t-tests or Mann–Whitney tests as appropriate for normal or nonnormal distribution of results, respectively. Categorical variables were analyzed by the Chi-square test. Adjusted analyses were performed using general linear model, analysis of variance (GLM ANOVA) entering all independent variables of interest simultaneously; no stepwise methods were used. Bonferroni adjustment of p values for multiple comparisons was used for subgroup comparisons. Partial Eta^2^ was calculated as an effect size measure of independent predictive variables in the model. Partial Eta^2^ represents the proportion of explained variance in dependent variable (serum creatinine concentration) by the independent predictor variable. Predicted values of serum creatinine concentration by the model were subtracted from measured values and these residuals were used for calculation of mean prediction error (normalized to observed creatinine concentration) and its confidence interval by Student's t-test. The probability level of <0.05 was considered statistically significant. Analyses were done using the IBM SPSS statistics application version 22 (IBM Corporation, USA).

## 3. Results

We were able to include 90 dialysis patients and 106 healthy controls with known creatinine values in the study sample. Details of selection process were given previously [14]. Demographic and clinical characteristics of study participants are given in Table 1. All dialysis patients were treated with high-flux dialysers without reuse and 52% of them were treated with online hemodiafiltration. Eighty-seven patients (97%) had established AV fistula or grafts (80 with native fistulas and 7 with grafts). Median residual urine output was 0.2 l/day (range 0-4 l/day).

Dialysis patients were divided into tertiles according to LBM to analyze the magnitude of differences in serum creatinine and associated variables. These results are shown in Table 2. There were statistically significant differences in serum creatinine, but also in age, height, and body mass across the tertiles of LBM. Going from lowest to highest tertile of lean mass, there were significant increases in height and body mass and decreases in age. Bonferroni post hoc tests revealed that when compared with the lowest tertile middle and highest tertile of LBM presented with 123 *μ*mol/l (p=0.09) and 192 *μ*mol/l (p=0.003) larger serum creatinine concentration. Results were similar in controls, except that there was no gradient in serum creatinine between first and second LBM tertiles and there was a significant difference in the range of 11-12 *μ*mol/l between lowest, middle, and highest tertiles of LBM (p<0.001).

**Figure 1:**
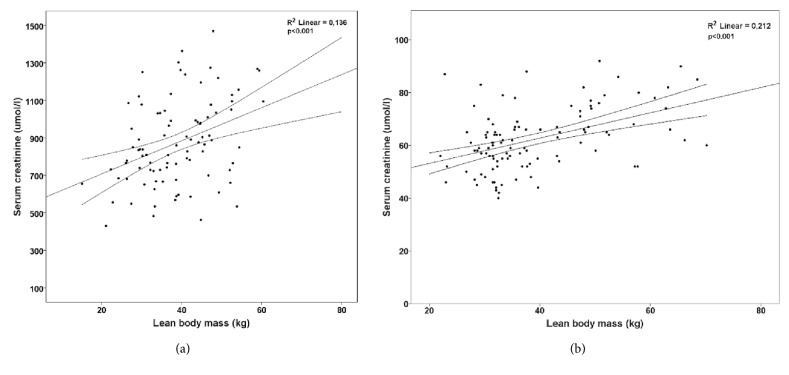
Scatter plot of relation between lean body mass and serum creatinine. Regression line with 95% CI for dialysis patients (a) and controls (b). For dialysis patients, the regression equation for serum creatinine is y=531 *μ*mol/l + 8.8 *μ*mol/l/kg x LBM (in kg). For controls the regression equation is 44 *μ*mol/l + 0.48 *μ*mol/l/kg x LBM (in kg).

In univariate analysis each kg of LBM predicted a rise in serum creatinine of 8.8 (95% CI 4.1-13.5) *μ*mol/l, p<0.001. LBM explained 13.6% of variability in serum creatinine. Univariate relation between LBM and serum creatinine is depicted in Figure 1.

When the model was supplemented with covariates of age, sex, height, LBM, and dialysis weekly dose (expressed in treatment time per week), explained variability rose to 20%; however LBM remained the only significant predictor of serum creatinine values. Similarly, the model was not significantly improved when handgrip strength and sit-to-stand test results representing the strength of upper and lower extremities were added as covariates to the model. The only two other predictors, significantly associated with serum creatinine values, were residual renal function expressed as daily urine volume and urea concentration. This final model explained 45% of variability in serum creatinine values and is shown in Table 3.

**Figure 2:**
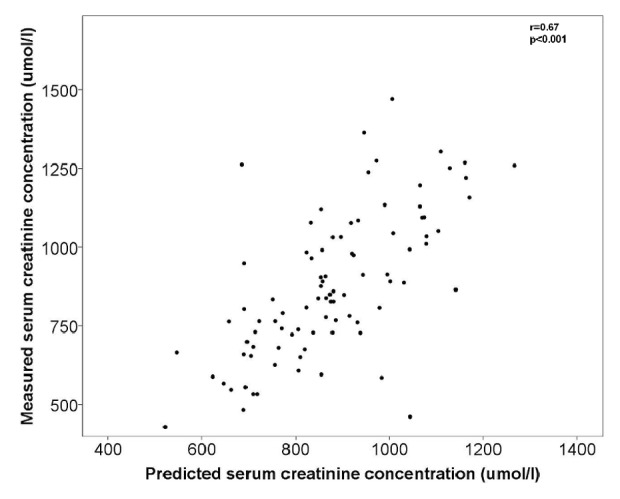
Predicted and measured serum creatinine values.

Predicted serum creatinine values based on this model with significant covariates LBM, residual diuresis, and urea had a good correlation with measured creatinine values with correlation coefficient of 0.67, p<0.001 (Figure 2). Mean prediction error was -4,1% (95% CI -8,8 to 0,7%).

Relation between LBM and serum creatinine in healthy controls is depicted in Figure 1(b). In healthy controls, LBM predicted 21.2% variability in serum creatinine with a coefficient of 0.48 (95% CI 0.3-0.66, p<0.001) *μ*mol/l/kg in unadjusted regression. The regression model was then supplemented with covariates of age, height, handgrip, and sit-to-stand performance and here only LBM and height predicted serum creatinine significantly, but there was a trend to a significant contribution by HGS and STS-10 performance. This model is shown in Table 4.

## 4. Discussion

In the present study we analyzed the relation of LBM as assessed by bioimpedance and serum creatinine values in ESRD patients. Results of this analysis have shown that crude unadjusted midweek predialysis serum creatinine values center around 770, 890, and 960 *μ*mol/l across the tertiles of LBM. Tertiles of LBM had similar median lean mass values in dialysis patients and controls with central tendencies of around 29-30 kg, 35-39 kg, and 48-51 kg in the lowest, middle, and highest tertile, respectively. Multivariate analysis enables the estimation of expected serum creatinine in dialysis patients by

(1) y=396+7.7  μmol/l/kg  x  LBMkg+12.9  μmol/mmol  x  ureammol/l−109  μmol/l2  x  residual  daily  urine  outputl

In unadjusted univariate regression LBM also showed a significant linear coefficient close to 8 *μ*mol/l/kg (exact value 8.8 *μ*mol/l/kg, 95% CI 4.1-13.5, and p<0.001). This quantitation allows for a quantitative evaluation of routine creatinine values when bioimpedance-assessed LBM is known. Further examination of possible underdialysis or sarcopenia problems is indicated when measured creatinine concentration is significantly higher or lower than predicted concentration based on LBM, respectively. Additionally, clinicians will be able to use this prediction to quickly estimate the discrepancy between predicted and observed creatinine values in predialysis CKD stage 5 cases with variable muscle mass when deciding on the need to start dialysis treatment.

We also tested the hypothesis that serum creatinine concentration may depend not only on the quantity of LBM but also on the quality of muscles measured through performance at handgrip and sit-to-stand tests. These two quick bedside tests may be the best choice to assess muscle performance in routine dialysis clinical practice [16]. However no association between these two test results and serum creatinine concentration could be found in our sample of dialysis patients. This result suggests that there is no additional explained variability in serum creatinine values by the muscle performance beyond the effects of muscle mass per se. Similarly, we observed no association of creatinine values with gender when the predictive model was adjusted for LBM. Since there was a difference in male/female ratio between patient and control groups this could potentially add some additional variability to the statistical models of patients and controls; however they were calculated separately and always adjusted for LBM, which is the only covariate physiologically expected to modify the association between gender and serum creatinine.

Bioimpedance technology is considered a possible alternative to dual energy X-ray absorptiometry for estimation of lean mass by European Working Group on Sarcopenia in Older People [22]. One of the drawbacks of bioimpedance spectroscopy to assess body composition is a possible overestimation of lean tissue when measurements are made on a nondialysis day due to overhydration effects [23, 24]. The amount of overhydration was however small in our sample: mean difference in extracellular fluid overhydration between dialysis patients and controls was +0.6l (95% CI 0.3-1l), so the overestimation of lean tissue in our analysis due to this methodological effect was small, if any. Another limitation is the lack of formal urea Kt/V values for dialysis treatment; however we were able to include in analysis a measure of residual renal function most accessible to clinical monitoring (residual daily diuresis) and the weekly time of dialysis treatment. In any case, addition of Kt/V in the prediction models of serum creatinine concentration could be theoretically expected to increase the total explained variability in serum creatinine concentrations but not modify the independent effect of LBM in the multivariate model. Previous work from another group revealed no significant association between urea Kt/V values and midweek predialysis serum creatinine (or even urea) concentrations so no significant improvement in our adjusted model can be expected by addition of urea Kt/V values [25]. Additional covariate which could further improve the predictive performance of adjusted model is protein intake or protein catabolic rate [25, 26]; unfortunately this variable was unavailable in the primary study protocol. Similarly, since this is a secondary analysis of a cross-sectional study aimed at establishing the magnitudes and predictive factors of physical performance deficits in uremia, no external validation cohort was available either. However this data may be used as a forerunner to a subsequent study with a focus on external validation of hereby established relations.

## 5. Conclusions

This cross-sectional case-control study was made to quantitatively describe the relation between LBM (assessed by bioimpedance) and serum creatinine values in hemodialysis patients. In adjusted analyses together with demographic and anthropometric covariates (age, sex, height, and body mass), LBM persisted as the only significant predictor of midweek predialysis serum creatinine concentration. Physical performance measures HGS and STS-10 did not improve prediction of serum creatinine. Each kg of LBM was associated with 7.7 *μ*mol/l increase in serum creatinine concentration. This data should help clinicians to better quantitatively evaluate observed creatinine concentrations of ESRD patients when bioimpedance derived LBM is available.

## Figures and Tables

**Table 1:** Demographic and clinical characteristics of study participants.

Parameter	Control group*∗*,†	Patients on HD*∗*	p
Sex (N male (%)/N female (%))	39(37%)/67(63%)	61(68%)/29(32%)	*<0.001*
Age (years)	55.5 (14.6)	55.2 (16)	0.87
BMI	26.4 (4.7)	26.1 (4.1)	0.57
Davies comorbidity grade 0/1/2 (N (%))	95(90%)/11(10%)/0	47(52%)/37(41%)/6(7%)	*<0.001*
Years of dialysis treatment	N/A	5 (1.7-11.5)	N/A
Weekly dialysis time (h)	N/A	14 (12-15)	N/A
Serum albumin (g/l)	44.8 (2.5)	40.8 (2.4)	*<0.001*
Serum urea (mmol/l)	N/A	24.8 (6.3)	N/A
Hemoglobin (g/l)	141 (12)	119 (12)	*<0.001*
Extracellular compartment overhydration (l)	0.4 (1)	1 (1.5)	*0.001*

BMI, body mass index; ESA, erythropoiesis-stimulating agent; HD, hemodialysis; PTH, parathyroid hormone; N/A, not applicable. *∗*Data are presented as mean (SD) or median (interquartile range) if not stated otherwise. ^†^Serum urea was not available in controls.

**Table 2:** Differences in serum creatinine, age, height, and body mass across tertiles of lean mass in dialysis patients and controls.

Parameter	1^st^ tertile	2^nd^ tertile	3^rd^ tertile	p
*Dialysis patients (N=89)*				
Lean body mass (kg)	29.4 (15.1-33.6)	38.7 (34-44)	47.9 (44.2-60.4)	*<0.001*
Age (years)	62.7 (14.8)	55 (17.1)	47.8	*0.001*
Height (cm)	161 (8)	170 (8)	174 (7)	*<0.001*
Body mass (kg)	67 (14)	75 (12)	81 (17)	*0.001*
Serum creatinine (*μ*mol/l)	770 (190)	893 (219)	962 (239)	*0.004*

*Controls (N=106)*				
Lean body mass (kg)	30.2 (22-31.9)	35.2 (32.1-40.1)	50.7 (43.1-70.2)	*<0.001*
Age (years)	61.4 (12.1)	54.5 (14.2)	50.7 (15.7)	*0.007*
Height (cm)	161 (5)	167 (7)	178 (6)	*<0.001*
Body mass (kg)	66 (11)	74 (16)	87 (16)	*<0.001*
Serum creatinine (*μ*mol/l)	59 (10)	58 (11)	70 (11)	*<0.001*

Data are presented as mean (SD) and for lean body mass as median (minimum-maximum).

**Table 3:** General linear model (ANOVA) for prediction of predialysis serum creatinine values.

Parameter	Coeff. B (SE)	95% CI	p	Partial Eta^2^
Intercept	396 (141)	115 - 676	*0.006*	0.09
Lean body mass (kg)	7.7 (2.2)	3.4 - 12.1	*0.001*	0.13
Residual diuresis (L/day)	-109 (26)	-160 to -57	*<0.001*	0.17
Age (years)	-1.4 (1.2)	-3.9 - 1.1	0.26	0.02
Urea (mmol/l)	12.9 (3.1)	6.7 - 19.1	*<0.001*	0.17

Model R^2^ 0.45 and p<0.001. Regression equation for serum creatinine on the basis of this model was y=396 + 7.7 *µ*mol/l/x LBM (kg) + 12.9 *µ*mol/mmol x urea (mmol/l) – 109 *µ*mol/l^2^ x residual daily urine output (l).

**Table 4:** General linear model (ANOVA) for prediction of serum creatinine values in healthy controls.

Parameter	Coeff. B (SE)	95% CI	p	Partial Eta^2^
Intercept	98 (30)	39 - 157	*0.001*	0.1
Lean body mass (kg)	0.58 (0.18)	0.23 – 0.94	*0.002*	0.1
Age (years)	0.004 (0.09)	-0.18 – 0.19	0.97	0
Height (cm)	-0.46 (0.21)	-0.88 to -0.05	*0.03*	0.05
Handgrip strength (kg)	0.36 (0.2)	-0.05 – 0.76	0.08	0.03
Sit-to-stand time (s)	0.5 (0.27)	-0.04 – 1.03	0.07	0.03

Model R^2^ 0.27 and *p<0.001*.

## Data Availability

Numerical data is available to interested readers upon request to the corresponding author of this article.
